# Predictors of high SARS-CoV-2 immunoglobulin G titers in COVID-19 convalescent whole-blood donors: a cross-sectional study in China

**DOI:** 10.3389/fimmu.2023.1191479

**Published:** 2023-06-14

**Authors:** Jingyun Tang, Humin Liu, Qing Wang, Xiaobo Gu, Jia Wang, Wenjun Li, Yinglan Luo, Yan Li, Lan Deng, Yue Luo, Xinman Du, Donglin Tan, Xuemei Fu, Xue Chen

**Affiliations:** ^1^ Blood Research Laboratory, Chengdu Blood Center, Chengdu, Sichuan, China; ^2^ Department of Blood Testing, Chengdu Blood Center, Chengdu, Sichuan, China; ^3^ Department of Blood Collection, Chengdu Blood Center, Chengdu, Sichuan, China; ^4^ Department of Blood Processing, Chengdu Blood Center, Chengdu, Sichuan, China

**Keywords:** SARS-CoV-2 IgG titer, convalescent plasma screening, COVID-19, Chinese, cross-sectional study, whole-blood donors

## Abstract

**Background:**

Demographic information has been shown to help predict high antibody titers of COVID-19 convalescent plasma (CCP) in CCP donors. However, there is no research on the Chinese population and little evidence on whole-blood donors. Therefore, we aimed to investigate these associations among Chinese blood donors after SARS-CoV-2 infection.

**Methods:**

In this cross-sectional study, 5,064 qualified blood donors with confirmed or suspected SARS-CoV-2 infection completed a self-reported questionnaire and underwent tests of SARS-CoV-2 Immunoglobulin G (IgG) antibody and ABO blood type. Logistic regression models were used to calculate odds ratios (ORs) for high SARS-CoV-2 IgG titers according to each factor.

**Results:**

Totally, 1,799 participants (with SARS-CoV-2 IgG titers≥1:160) had high-titer CCPs. Multivariable analysis showed that a 10-year increment in age and earlier donation were associated with higher odds of high-titer CCP, while medical personnel was associated with lower odds. The ORs (95% CIs) of high-titer CCP were 1.17 (1.10–1.23, p< 0.001) and 1.41 (1.25-1.58, p< 0.001) for each 10-year increment in age and earlier donation, respectively. The OR of high-titer CCP was 0.75 (0.60-0.95, p = 0.02) for medical personnel. Female early donors were associated with increased odds of high-titer CCP, but this association was insignificant for later donors. Donating after 8 weeks from the onset was associated with decreased odds of having high-titer CCP compared to donating within 8 weeks from the onset, and the HR was 0.38 (95% CI: 0.22-0.64, p <0.001). There was no significant association between ABO blood type or race and the odds of high-titer CCP.

**Discussion:**

Older age, earlier donation, female early donors, and non-medical-related occupations are promising predictors of high-titer CCP in Chinese blood donors. Our findings highlight the importance of CCP screening at the early stage of the pandemic.

## 1 Introduction

The ongoing coronavirus disease (COVID-19) pandemic, caused by severe acute respiratory syndrome coronavirus 2 (SARS-CoV-2), remains a major threat to global health (1). Convalescent plasma therapy has emerged as a safe and potentially beneficial supplementary treatment for COVID-19 patients through direct viral neutralization and Fc-dependent functions (2). Despite the highly controversial efficacy of COVID-19 convalescent plasma (CCP) for COVID-19 inpatients, early utilization of high-titer CCPs (neutralizing titer >160) is more likely to be effective in reducing mortality (3). In addition, CCP transfusion is recommended by the American Association of Blood Banks (AABB) for high-risk early-stage COVID-19 outpatients, immunocompromised patients, and those with undetectable antibody titers (4).

During the pandemic, SARS-CoV-2 has been observed to mutate constantly, thus leading to a weakening of the protective effects offered by vaccines against infection (5). Though small molecule antivirals remain effective, most monoclonal antibody therapies have become ineffective against the Omicron sublineages due to immune escape (6). In contrast, CCP, which contains antibodies with diverse specificities and represents all isotypes, may be an effective antibody therapy for immunocompromised patients (7).

The neutralizing antibody titer is a critical parameter of CCP treatment (2, 8). Many countries have developed strategies for CCP screening and defined high-titer plasma based on various serological biomarkers such as titers of neutralizing antibodies, IgG antibodies, and total antibodies (2, 4, 9–11). The Clinical treatment scheme of COVID-19 convalescent plasma (pilot 3^rd^ edition) (12) specifies that CCP (high titer) in China should test positive after 160-fold dilution of COVID-19 serum/plasma IgG antibody or 320-fold dilution of total antibody using ELISA or chemiluminescence.

Previous studies identified several factors that may predict high-titer antibodies in CCP donors, including age (13–17), gender (13–15, 18), race/ethnicity (13, 19), BMI (20), complications (15), SARS-CoV-2 infection symptoms (14, 16), illness severity (15), and time after infection (13, 16, 21). Identifying these predictive factors of high-titer CCP can assist in streamlining screening, allowing for more efficient fulfillment of clinical demands while also reducing screening costs.

CCPs can be collected not only via apheresis directly but also from donated whole blood, which can widen the source of CCPs while reducing plasma waste. However, there is no research on CCP screening in Chinese whole-blood donors. With the dramatic changes in COVID-19 prevalence in China following the removal of all COVID-19-related restrictions and the continuous mutation of SARS-CoV-2, the levels of SARS-CoV-2 antibodies may be influenced by various factors, including widespread vaccination (22). Therefore, we aim to identify potential predictive factors of high-titer CCP (including donors’ age, sex, ABO blood type, race/ethnicity, occupation, and donation time) among Chinese whole-blood donors who have recovered from COVID-19 infection in Chengdu. All of the aforementioned information is routinely collected during blood donation. The findings of this study will contribute to identifying the optimal donors and developing more effective CCP screening strategies in Chengdu and other regions in light of the new pandemic context.

## 2 Materials and methods

### 2.1 Study design and ethics

This single-center, cross-sectional study was approved by The Ethics Committee of Chengdu Blood Center (No. CDSXYZX-LSY202301), and the written informed consents were signed by all participants.

### 2.2 Population

**Figure 1:**
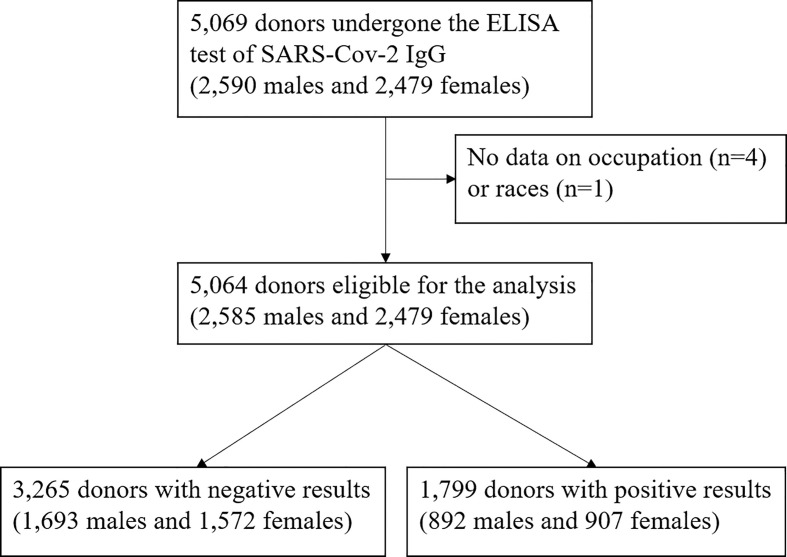
Flow chart.

The study was conducted in Chengdu from 25^th^ December 2022 to 15^th^ February 2023, approximately 3-10 weeks after implementing an adjusted anti-epidemic policy on 7^th^ December 2022. Blood donors with confirmed or suspected SARS-CoV-2 infection were screened, and whole blood was collected at least seven days after their last positive results of the nucleic acid test or antigen test. All donors satisfied the standard eligibility criteria for blood donation ruled by the 2019 Technical Operating Regulation for Blood Station (23) (for example, no history or high-risk factors of transfusion-transmitted infections such as HIV, hepatitis B virus, hepatitis C virus, and Treponema pallidum). The donation dates of donors were recorded, and their basic demographic information (including age, sex, race, and occupation) was obtained from the Blood Donor Registration Form, a self-reported questionnaire for all eligible blood donors. A total of 5,069 qualified donors underwent tests of ABO blood types and SARS-CoV-2 IgG antibodies. Individuals without information on race (n=1) or occupation (n=4) were excluded. Finally, 5,064 blood donors were included in our study (**Figure 1**). In our study, 397 out of the 5064 blood donors completed an additional questionnaire (from 4^th^ January to 14^th^ February 2023), 393 of which replied to the following question: How long has it been since your onset? (If you were asymptomatic, please choose the diagnosis time): 1) Within 2 weeks; 2) 2-3 weeks (including 2 weeks); 3) 3-4 weeks (including 3 weeks); 4) 4-8 weeks (including 4 weeks); 5) 8 weeks to 6 months (including 8 weeks); 6) 6 months and above.

### 2.3 Measurements of ABO blood type and SARS-CoV-2 IgG antibody titer

Each donor’s ABO blood type was determined, and approximately 5 mL blood sample was collected in EDTA (Ethylene Diaminete Tracetic Acid) tubes. After excluding unqualified samples such as lipid blood or hemolysis blood, the remained blood samples were centrifuged at 3000 r/min for 10 minutes.

Samples were tested using the commercial Wantai’ SARS-CoV-2 IgG antibody enzyme-linked immunosorbent assay (ELISA) after being diluted 160-fold. The validation of substituting the neutralization test has been reported previously (24). Testing was performed on an ML-FAME 24/30 full-automatic enzyme immunoassay analyzer (Hamilton Bonaduz AG, Switzerland) according to the instruction provided. The 100uL kit-specific sample dilution buffer was added to each well except for the wells for blank, positive, or negative control. Then, 10 uL diluted samples, 100 uL positive control, or negative assay control were added to the corresponding wells, followed by a 30-minute incubation at 37°C. Plates were washed five times with kit-specific wash buffer. Anti-human IgG-HRP-conjugated detection antibodies were added, followed by another round of incubation and plate-washing process. After a 15-minute incubation in the dark with tetramethylbenzidine, sulfuric acid was added. The absorbance at 450 nm was measured within 10 minutes after the reaction was terminated.

The positive cutoff values were calculated using the formula: cutoff =0.16+xNC, where xNC is the average OD-value of triplicate negative controls (OD-values less than 0.03 was calculated as 0.03). Samples with OD-values equal to or greater than the positive cutoff values were reported as positive.

### 2.4 Statistical analysis

Samples with positive SARS-CoV-2 IgG (titers ≥1:160) were designated as high-titer CCPs. The characteristics of participants with or without high-titer CCP, as well as the overall participant pool, were presented as means ± SDs or proportions (number of cases). We investigated potential predictors of high-titer CCP including age (per 10 years), age groups (18-30 years old, 31-40 years old, 41-50 years old, and 51-60 years old), sex (males or females), race (Han-individuals or the others), occupation (medical personnel or the others), donation time [early (within 3-5 weeks since the policy adjustment) or late (5-10 weeks after the policy adjustment)] and ABO blood type (A, B, AB, and O). Logistic regression analyses were conducted for each variable to estimate the odds ratios (ORs) and 95% confidence intervals (CIs) for high-titer CCP. Besides the unadjusted model, we applied a multivariable model that adjusted each variable for all the other potential predictive factors mentioned above. Age was treated as a continuous variable in all the models, except for the models specifically designed for analyzing age groups. Furthermore, we performed further analyses stratified by donation time, and all the aforementioned factors were included in the stratified multivariable models, except for donation time. Multiplicative interactions related to donation time were assessed, and p-values for these interactions were calculated by incorporating a cross-product term for donation time with each other variable into the model. SAS version 9.4 was used for all statistical analyses. Reported p-values were two-sided, with values less than 0.05 considered statistically significant.

## 3 Results

In our study, 1,799 out of 5,064 (35.5%) participants (2,585 males and 2,479 females) were defined with a high-titer CCP. We summarized the characteristics of participants with or without high-titer CCP and the overall participant pool in **Table 1**. The mean ages were 37.1, 38.7, and 37.7 years, respectively. Corresponding percentages of medical personnel among each population were 9%, 6%, and 8%. Early donors account for 47%, 55%, and 50% of each population. Individuals with a high-titer CCP were more likely to be older, non-medical-related persons, and donate blood at the early stage of the pandemic compared to those without a high-titer CCP. The prevalences of A, B, AB, and O blood groups were 32%, 25%, 12%, and 32%, respectively. Han individuals account for 97% of the whole population. There is no significant inter-group difference in sex, ABO blood type, or race.

**Table 1:** Participants’ characteristics according to SARS-CoV-2 IgG titers.

	SARS-CoV-2 IgG titers< 1:160	SARS-CoV-2 IgG titers ≥1:160	Overall
**Number of cases, % (n)**	64 (3265)	36 (1799)	5064
**Age, year**	37.1 ± 10.2	38.7 ± 10.6‡	37.7 ± 10.3
18-30, % (n)	30 (990)	26 (469) †	29 (1459)
31-40, % (n)	31 (998)	29 (517)	30 (1515)
41-50, % (n)	28 (916)	28 (505)	28 (1421)
51-60, % (n)	11 (361)	17 (308)‡	13 (669)
**Males, % (n)**	52 (1693)	50 (892)	51 (2585)
ABO blood type			
A, % (n)	32 (1031)	33 (598)	32 (1629)
B, % (n)	25 (806)	24 (435)	25 (1241)
AB, % (n)	12 (387)	11 (197)	12 (584)
O, % (n)	32 (1041)	32 (569)	32 (1610)
**Race, Han individuals, % (n)**	97 (3166)	97 (1740)	97 (4906)
**Occupation, medical personnel, % (n)**	9 (281)	6 (114)*	8 (395)
**Donated during the early stage, % (n)**	47 (1547)	55 (995)‡	50 (2542)

* p<0.05; †p<0.01; ‡p<0.001. P for all the variables other than age are age-adjusted. The age-adjusted p-values were estimated by the regression method (linear regression for continuous variables and logistic regression for categorical variables).

Means ± standard deviation for continuous variables and percentages (numbers of cases) for categorical variables.

The associations between all variables of interest and high-titer CCP are shown in **Table 2**. A 10-year increment in age and an earlier donation were observed to be associated with higher odds of high-titer CCP, while medical personnel was associated with lower odds. The corresponding ORs were 1.17 (95% CI: 1.10–1.23, p< 0.001), 1.41 (95% CI: 1.25-1.58, p< 0.001), and 0.75 (95% CI: 0.60-0.95, p = 0.02). The multivariable OR (95% CI) of high-titer CCP for males is 0.91 (0.81-1.02) compared with females. No association of ABO blood type or race was observed with the odds of high-titer CCP. In comparison to Type A, the ORs (95% CI) were 0.93 (0.80-1.09) for Type B, 0.92 (0.75-1.12) for Type AB, and 0.96 (0.83-1.11) for Type O, respectively. The OR (95% CI) for Han individuals compared to the others was 0.86 (0.62-1.20).

**Table 2:** Odds Ratios (95% CIs) of high-titer CCPs according to potential predictive factors.

	Unadjusted Odds Ratios (95% CIs)	Multivariable Odds Ratios (95% CIs)*	*p*
Total donors, n	5064		
**Age (per 10-year increment)**	1.16 (1.10-1.23)	1.17 (1.10-1.23)	<0.001
**Age groups**			
31-40 vs. 18-30	1.09 (0.94-1.27)	1.08 (0.93-1.27)	0.01
41-50 vs. 18-30	1.16 (1.00-1.36)	1.16 (0.99-1.36)	0.24
51-60 vs. 18-30	1.80 (1.49-2.17)	1.82 (1.50-2.20)	<0.001
**Sex (males vs. females)**	0.91 (0.81-1.03)	0.91 (0.81-1.02)	0.10
**ABO blood type**			
B vs. A	0.93 (0.80-1.09)	0.93 (0.80-1.09)	0.71
AB vs. A	0.88 (0.72-1.07)	0.92 (0.75-1.12)	0.62
O vs. A	0.94 (0.82-1.09)	0.96 (0.83-1.11)	0.92
**Race (Han individuals vs. the others)**	0.92 (0.66-1.28)	0.86 (0.62-1.20)	0.37
**Occupation (medical personnel vs. the others)**	0.72 (0.57-0.90)	0.75 (0.60-0.95)	0.02
**Donation time (early vs. late), %**	1.37 (1.22-1.54)	1.41 (1.25-1.58)	<0.001
Earlier donors†, n	2542		
**Age (per 10-year increment)**	1.16 (1.08-1.26)	1.16 (1.07-1.25)	<0.001
**Age groups**			
31-40 vs. 18-30	1.11 (0.90-1.36)	1.09 (0.89-1.34)	0.06
41-50 vs. 18-30	1.23 (0.99-1.52)	1.21 (0.97-1.49)	0.69
51-60 vs. 18-30	1.84 (1.40-2.40)	1.80 (1.37-2.36)	<0.001
**Sex (males vs. females)**	0.83 (0.71-0.98)	0.84 (0.72-0.99)	0.04
**ABO blood type**			
B vs. A	0.96 (0.78-1.18)	0.96 (0.78-1.18)	0.88
AB vs. A	0.96 (0.71-1.28)	0.95 (0.71-1.27)	0.82
O vs. A	0.97 (0.80-1.19)	0.98 (0.80-1.20)	0.92
**Race (Han individuals vs. the others)**	1.11 (0.69-1.78)	1.04 (0.64-1.67)	0.88
**Occupation (medical personnel vs. the others)**	0.91 (0.68-1.23)	0.93 (0.69-1.26)	0.65
Later donors†, n	2522		
**Age (per 10-year increment)**	1.19 (1.10-1.29)	1.17 (1.08-1.27)	<0.001
**Age groups**			
31-40 vs. 18-30	1.09 (0.87-1.38)	1.06 (0.84-1.34)	0.08
41-50 vs. 18-30	1.15 (0.92-1.45)	1.11 (0.88-1.39)	0.24
51-60 vs. 18-30	1.88 (1.44-2.46)	1.80 (1.37-2.35)	<0.001
**Sex (males vs. females)**	1.02 (0.86-1.21)	0.98 (0.82-1.16)	0.78
**ABO blood type**			
B vs. A	0.88 (0.70-1.11)	0.90 (0.71-1.13)	0.60
AB vs. A	0.86 (0.65-1.13)	0.92 (0.69-1.21)	0.84
O vs. A	0.93 (0.76-1.14)	0.93 (0.76-1.15)	0.94
**Race (Han individuals vs. the others)**	0.76 (0.48-1.20)	0.73 (0.46-1.15)	0.18
**Occupation (medical personnel vs. the others)**	0.52 (0.36-0.74)	0.56 (0.39-0.81)	0.002

* Adjusted for all the other variables listed in tables for each variable. Age was treated as a continuous variable in all the models, except for the models specifically designed for analyzing age groups. Time-stratified analyses were adjusted for all variables other than the donation time.

† Earlier donors and later donors were those who donated within 3-5 weeks or 5-10 weeks after the policy adjustment, respectively.

‡ p-values were calculated for multivariable models.

We further performed time-stratified analyses since there was an interaction between donation time and occupation (p = 0.02) (data not shown). P-values for interaction were larger than 0.05 for all other factors interacted by donation time and 0.12 for sex. The positive association between age and high-titer CCP was observed in both earlier donors and later donors, and the corresponding HRs were 1.16 (1.07-1.25) and 1.17 (1.08-1.27) per 10-year age increment. However, the negative association between medical personnel and high-titer CCP was confined to later donors, and the HRs (95% CIs) were 0.93 (0.69-1.26) among earlier donors and 0.56 (0.39-0.81, p = 0.002) among later donors. In addition, we found male borderline associated with lower odds of high-titer CCP among earlier but not later donors, and the HRs were 0.84 (95% CI: 0.72-0.99, p = 0.04) and 0.98 (95% CI: 0.82-1.16), respectively. No significant association of ABO blood type or race with high-titer CCP was observed in time-stratified analyses.

There is a strong correlation between the time interval from symptom onset to blood donation and donation time (**Supplementary Table 1**). The Spearman correlation coefficient between donation time and time intervals divided into 6, 3, and 2 groups were -0.60, -0.62, and -0.73, respectively, all p-values were less than 0.001. **Supplementary Table 2** showed the association between time from the onset to donation and high-titer CCP. Few individuals donated 2-3 weeks after the onset or 6 months later. In reference to those who donated 4-8 weeks (including 4 weeks) after the COVID-19 onset, those who donated within 4 weeks had higher odds while those who donated 8 weeks after the onset had lower odds to have high-titer CCP. The corresponding HRs were 2.69 (95% CI: 1.51-4.78, p <0.001) and 1.03 (95% CI: 0.61-1.72, p = 0.06). Donating after 8 weeks from the onset was associated with a decreased likelihood of having high-titer CCP compared to donating within 8 weeks from the onset, and the HR was 0.38 (95% CI: 0.22-0.64, p <0.001).

## 4 Discussion

To our knowledge, this study represents the first to investigate potential predictive factors of high-titer CCP in Chinese whole-blood donors. In this cross-sectional study of 5,064 whole-blood donors who have recovered from confirmed or suspected COVID-19 infection, we found that older age, earlier donation, and non-medical-related occupations were associated with a higher prevalence of high-titer CCP. Of note, the negative association between medical personnel and high-titer CCP was confined to later donors, and females were associated with increased odds of high-titer CCP among earlier but not later donors. In addition, our findings revealed a negative association between a longer time interval from COVID-19 infection onset to donation and the likelihood of high-titer CCP.

The CCP screening was carried out 3-10 weeks after the Chinese government lifted all COVID-19-related restrictions. The blood donors had received widespread vaccination. Our findings can aid in identifying the optimal donors and developing improved CCP screening strategies in Chengdu and other regions affected by the pandemic. By narrowing the screening scope, not only can screening efficiency be improved, but costs and labor can also be saved. Furthermore, our findings could provide additional evidence of diverse antibody responses to SARS-CoV-2 among different populations and time periods.

Numerous studies have reported positive associations between older age and high-titer CCP (13–16). In a longitudinal study of 52,240 US CCP donors, older donors had significantly higher initial SARS-CoV-2 antibody titers compared to their younger counterparts (*p*< 0.0001), and the antibody levels persisted for a longer period in donors aged 55 to 66 years compared to other age groups (*p* = 0.0004) (13). Similarly, another longitudinal study of 2,082 CCP donors in the Netherlands reported a positive association between age and peak IgG concentration, with a fixed effect point estimate (95% CI) of 0.128 (0.100-0.157) per 10 years increment in age (14). Consistent with these findings, our current study also observed a similar association between age and CCP titers among both earlier and later donors.

Previous studies have indicated that SARS-CoV-2 antibodies decline in patients or CCP donors over time (13, 14), with time (measured in days) since symptom onset being a predictor of lower antibody titers (estimate: 0.97, 95% CI: 0.995–0.998, p<0.001) (16). Our findings are consistent with these previous studies, as we observed that earlier donations were associated with increased odds of having high-titer CCP. This underscores the importance of early CCP screening.

Health or social care workers were associated with higher SARS-CoV-2 antibody titers in seropositive participants compared to non-frontline workers, regardless of disease severity, possibly due to their higher environmental exposure (OR:1.19,95% CI: 1.07–1.33, p = 0.001) (25). However, an opposite association was observed among later donors. This may be attributed to the high prevalence in the general population, which could reduce the inter-group difference in environmental exposure. In addition, medical personnel were vaccinated earlier and possibly infected earlier than the general population, and antibody responses triggered by vaccination and infection weakened over time (13, 26, 27).

Numerous studies have investigated the association between sex and high-titer CCP, with some reporting a positive association of males (15, 19, 28) and some finding no association (17). In our study, female donors had higher odds of high-titer CCP than male donors at the early stage of the pandemic, whereas no sex-specific association was observed among later donors. Sex‐specific differences in the immune response to COVID-19 infection and vaccines, as well as clinical outcomes, have been reported (29, 30). A longitudinal study of 52,240 US CCP donors showed that females had higher initial antibody titers, but experienced a faster decline over time compared to males (13), which may partially explain our findings. However, another study of 2,082 Netherlands CCP donors reported that antibodies declined faster in male donors than in female donors (14). These controversial results may be attributed to differences in assay method, observation time, and study population (different in age, race, and disease severity). Another potential reason for our findings may be related to the use of whole-blood sources, as some potential high-titer male blood donors in our study population may be excluded for severe COVID-19 infection. It has been extensively recognized that males have a higher risk of severe COVID-19 infection and mortality than females (31). Additionally, the severity of COVID-19 infection has been associated with increased antibody levels (32, 33), and may slow down the decline of anti-spike IgG antibodies (34). Further studies are needed to better understand the underlying mechanism and explore the efficacy of corresponding CCPs.

The associations between ABO blood types with high-titer CCPs have shown considerable variation across studies. In different populations, higher titers in CCPs were associated with A or AB individuals compared to O or B individuals (35), B individuals compared with O individuals (36), AB individuals compared to the others (16), and A individuals compared with O individuals (37). However, like many others (20, 38–40), no significant association between ABO blood types and high titer was observed in our study. These divergent results may be caused by differences in age, race, sample size, testing methods, and definition of outcomes. Additionally, different susceptibilities and consequent variations in the timing of infection among individuals with different blood types may provide another explanation (41).

Although race was identified as a potential predictor of high-titer CCP (13, 19, 25), we were unable to confirm an association due to the small sample size of non-Han individuals in our study.

Our study is the first to investigate the predictors of high-titer CCP in Chinese blood donors. Furthermore, our study has several strengths, including the use of whole-blood source, standard measurements of both SARS-CoV-2 IgG titers and potential predictive factors, all within the context of the latest pandemic. The study has several limitations. Firstly, although we adjusted for some potential predictive factors, the influence of some other important factors, such as vaccination status and duration from symptom onset to blood collection, cannot be ruled out. Secondly, instead of testing all infected or suspected samples, we preferentially selected individuals who donated 300 or 400 ml but not 200 ml of whole blood. Additionally, we accounted for ABO blood types to meet clinical needs in the later stage, which may have introduced selection bias. Thirdly, the single-centered cross-sectional design and the limited sample size may have restricted the generalizability of our findings. Therefore, multi-center and longitudinal studies with larger sample sizes and more detailed time-varying data are needed to validate our findings.

In summary, our study suggests that older age, earlier donation, female early donors, and non-medical-related persons in the late stage are promising predictors of high-titer CCP in Chinese blood donors in Chengdu. The findings highlight the importance of early CCP screening. However, further research with larger sample sizes and more diverse populations is warranted to validate these results.

## Data availability statement

The raw data supporting the conclusions of this article will be made available by the authors, without undue reservation.

## Ethics statement

The studies involving human participants were reviewed and approved by the Ethics Committee of Chengdu Blood Center (No. CDSXYZX-LSY202301). The patients/participants provided their written informed consent to participate in this study.

## Author contributions

XC and XF designed this study. QW, XG, JW, YaL, and LD collected data; Statistical analysis: JT analyzed results and made the figures. HL, WL, and YiL measured blood samples. JT and XC drafted of the manuscript. All authors revised the manuscript. All authors have read and approved the final manuscript. All authors agreed to be accountable for the content of the work.

## Supplementary material

Supplementary Table 1 Time intervals from onset to donation according to donating time (earlier or later).

Supplementary Table 2 Odds Ratios (95% CIs) of high-titer CCPs according to time intervals from onset to donation.
