# Corrigendum: Predictors of high SARS-CoV-2 immunoglobulin G titers in COVID-19 convalescent whole-blood donors: a cross-sectional study in China

**DOI:** 10.3389/fimmu.2023.1327039

**Published:** 2023-11-07

**Authors:** Jingyun Tang, Humin Liu, Qing Wang, Xiaobo Gu, Jia Wang, Wenjun Li, Yinglan Luo, Yan Li, Lan Deng, Yue Luo, Xinman Du, Donglin Tan, Xuemei Fu, Xue Chen

**Affiliations:** ^1^ Blood Research Laboratory, Chengdu Blood Center, Chengdu, Sichuan, China; ^2^ Department of Blood Testing, Chengdu Blood Center, Chengdu, Sichuan, China; ^3^ Department of Blood Collection, Chengdu Blood Center, Chengdu, Sichuan, China; ^4^ Department of Blood Processing, Chengdu Blood Center, Chengdu, Sichuan, China

**Keywords:** SARS-CoV-2 IgG titer, convalescent plasma screening, COVID-19, Chinese, cross-sectional study, whole-blood donors

In the published article, there was an error. A mistake on method description was introduced during the English language editing process prior to manuscript submission.

A correction has been made to the section **Materials and Methods**, subsection *2.4 Statistical Analysis*, paragraph 1. This sentence previously stated:

“A two-way ANOVA was used to analyze the interaction effects and to calculate p-values for interaction.”

The corrected sentence appears below:

“Multiplicative interactions related to donation time were assessed, and p-values for these interactions were calculated by incorporating a cross-product term for donation time with each other variable into the model.”

The authors apologize for this error and state that this does not change the scientific conclusions of the article in any way. The original article has been updated.

