# Supplementary material for: Predictors of high SARS-CoV-2 immunoglobulin G titers in COVID-19 convalescent whole-blood donors: a cross-sectional study in China
Source: Front Immunol. 2023 Jun 14;14:1191479. doi: 10.3389/fimmu.2023.1191479 (PMC10303911; doi:10.3389/fimmu.2023.1191479)
Supplement: Supplementary Table 1 — Time intervals from onset to donation according to donating time (earlier or later). [file Table_1.docx]

| **Supplementary Table 1. Time intervals from onset to donation according to donating time (earlier or later)** | | | |  |
| --- | --- | --- | --- | --- |
|  | Earlier donors | Later donors | Overall |  |
| **Time intervals from onset to donation ( < 2 weeks, 2-3 weeks, 3-4 weeks, 4-8 weeks, 8 weeks-6 months, >= 6 months)*** | | | |  |
| less than 2 weeks, % (n) | 6.1(6) | 1.7(5) | 2.8(11) |  |
| 2-3 weeks (including 2 weeks), % (n) | 8.1(8) | 0.3(1) | 2.3(9) |  |
| 3-4 weeks (including 3 weeks), % (n) | 56.6(56) | 1.0(3) | 15(59) |  |
| 4-8 weeks (including 4 weeks), % (n) | 25.3(25) | 55.1(162) | 47.6(187) |  |
| 8 weeks- 6 months (including 8 weeks), % (n) | 1.0(1) | 39.8(117) | 30(118) |  |
| 6 months or above | 3.0(3) | 2.0(6) | 2.3(9) |  |
| **Time intervals from onset to donation ( < 4 weeks, 4-8 weeks, >=8 weeks)†** | | | |  |
| less than 4 weeks, % (n) | 70.7(70) | 3.1(9) | 20.1(79) |  |
| 4-8 weeks (including 4 weeks), % (n) | 25.3(25) | 55.1(162) | 47.6(187) |  |
| 8 weeks or above, % (n) | 4.0(4) | 41.8(123) | 32.3(127) |  |
| **Time intervals from onset to donation ( < 4 weeks, >= 4 weeks)‡** | | | |  |
| less than 4 weeks, % (n) | 70.7(70) | 3.1(9) | 20.1(79) |  |
| 4 weeks or above, % (n) | 29.3(29) | 96.9(285) | 79.9(314) |  |
| * Spearman correlation coefficient between time interval and donation time = -0.60, p< 0.001; † Spearman correlation coefficient between time interval and donation time = -0.62, p< 0.001; ‡ Spearman correlation coefficient between time interval and donation time = -0.73, p< 0.001. | | | |  |
|  |  |  |  |  |
|  |  |  |  |  |
