# Supplementary material for: Predictors of high SARS-CoV-2 immunoglobulin G titers in COVID-19 convalescent whole-blood donors: a cross-sectional study in China
Source: Front Immunol. 2023 Jun 14;14:1191479. doi: 10.3389/fimmu.2023.1191479 (PMC10303911; doi:10.3389/fimmu.2023.1191479)
Supplement: Supplementary Table 2 — Odds Ratios (95% CIs) of high-titer CCPs according to time intervals from onset to donation. [file Table_2.docx]

| **Supplementary Table 2. Odds Ratios (95% CIs) of high-titer CCPs according to time intervals from onset to donation** | | | |
| --- | --- | --- | --- |
|  | Numbers of donors (numbers of high-titer donors) | Unadjusted Odds Ratios (95% CIs) | Multivariable Odds Ratios (95% CIs)* |
| **Time interval (6 groups), n** | 394(122) |  |  |
| less than 2 weeks | 11(8) | 7.11(1.82-27.86) | 9.32(2.28-38.18) |
| 2-3 weeks (including 2 weeks) | 9(2) | 0.76(0.15-3.79) | 0.75(0.15-3.82) |
| 3-4 weeks (including 3 weeks) | 59(27) | 2.25(1.23-4.12) | 2.60(1.37-4.91) |
| 4-8 weeks (including 4 weeks) (Ref.) | 187(51) | 1 | 1 |
| 8 weeks- 6 months (including 8 weeks) | 118(31) | 0.95(0.56-1.6) | 1.00(0.59-1.69) |
| 6 months or above | 9(3) | 1.33(0.32-5.53) | 1.58(0.36-6.93) |
| **Time interval (3 groups)** |  |  |  |
| less than 4 weeks | 79(27) | 2.35(1.36-4.06) | 2.69(1.51-4.78) |
| 4-8 weeks (including 4 weeks) (Ref.) | 187(51) | 1 | 1 |
| 8 weeks or above | 127(34) | 0.98(0.59-1.62) | 1.03(0.61-1.72) |
| **Time interval (2 groups)** |  |  |  |
| less than 4 weeks (Ref.) | 79(37) | 1 | 1 |
| 4 weeks or above | 314(85) | 0.42(0.25-0.70) | 0.38(0.22-0.64) |
| * Adjusted for age, sex, ABO blood type, race, and occupation. | | | |
| † *p*-values were calculated for multivariable models. | | | |
